# Analysis of perioperative problems related to intraocular Implantable Collamer Lens (ICL) implantation

**DOI:** 10.1007/s10792-022-02355-w

**Published:** 2022-06-22

**Authors:** Hao Zhang, Rui Gong, Xiaolan Zhang, Yingping Deng

**Affiliations:** Department of Ophthalmology, West China Hospital, Sichuan University, No.37, Guoxue Xiang, Chengdu, 610041 Sichuan China

**Keywords:** Implantable Collamer Lens, Surgery, Perioperative problem, Treatment

## Abstract

**Purpose:**

To introduce a modified procedure of ICL implantation, to summarize the perioperative problems and their corresponding treatment after myopia correction with Implantable Collamer Lens (ICL), and to compare the difference of complications between the no-hole ICL and hole ICL.

**Methods:**

We searched all articles on ICL-related perioperative problems and their corresponding treatment in Scopus, Embase, PubMed and Web of Science databases for the last 22 years.

**Results:**

ICL implantation is safe, effective, stable and predictable in the correction of myopia, hyperopia and astigmatism, but can also cause a series of perioperative problems, including intraoperative and postoperative complications.

**Conclusion:**

There are many kinds of complications related to ICL, but the common intraoperative and postoperative complications mainly include abnormality of arch height, abnormal position of ICL, loss of corneal endothelial cells and corneal decompensation, high intraocular pressure and secondary glaucoma, cataract and night vision symptoms. Compared with ICL without central pore, the incidence of complications such as loss of corneal endothelial cells and corneal decompensation, high intraocular pressure and secondary glaucoma and cataract was relatively lower in central hole ICL, while postoperative complications such as night vision symptoms were obvious.

## Introduction of ICL process

The brief procedures of surgery we recommend are as follows: After topical anesthesia, a 2.7 mm or 3 mm superior clear corneal main incision is created. The anterior chamber was filled with viscoelastic agent. The ICL was inserted through the main incision with the use of an injector cartridge (STAAR Surgical, Switzerland) and was placed in the posterior chamber by gently tucking the footplates beneath the iris. The viscoelastic agent was suctioned out of the anterior chamber with irrigation/aspiration (IA) surgery handpiece. No preoperative or intraoperative peripheral iridectomy was performed in any case. After surgery, the patient's intraocular pressure was monitored for 2–4 h, and topical anti-inflammatory and infection prevention regimens were prescribed. Different surgeons and different hospitals have different topical anti-inflammatory and infection prevention regimens. In our hospital, it included 0.3% Tobramycin and 0.1% Dexamethasone ophthalmic suspension (Tobramycin and Dexamethasone Eye Drops, s.a. ALCON-COUVREUR n.v.; four times per day and cut back once a week for 4 weeks), Bromfenac Sodium Hydrate Ophthalmic Solution (Senju Pharmaceutical Co., Ltd., Japan; two times per day for 4 weeks).

The traditional incision is in the temporal side of the cornea, but we recommend a superior incision, because the vertically fixated ICL through a superior incision achieved good results without significant complications. Considering that younger patients requiring ICL surgery tend to have with-the-rule astigmatism, this surgical technique may be a viable option for reducing astigmatism without using toric ICL (TICL) [1]. The normal process is that viscoelastic agent will be first injected into the anterior chamber followed by ICL implantation. However, it was reported that reversing the order of the two had no effects on the corneal endothelial cell count. Additionally, the implantation of an intraocular lens prior to injecting OVDs reduces the operation time and lowers the rate of IOP rise in the early postoperative period, making it safe and effective for ICL implantation [2]. Therefore, we recommend the modified surgical procedure.

## Summary and treatment of intraoperative complications of ICL

### Operation injury

Surgical injury can lead to conjunctival or intraocular hemorrhage, corneal epithelial defects, corneal edema, descemet membrane detachment of the cornea, anterior chamber angle injury, and traumatic cataract, so the surgeon's learning curve is considered to be a risk factor for postoperative cataract formation. Traumatic cataract is the most serious consequence of surgical injury. For non-central hole ICL implantation, when performing intraoperative peripheral iridectomy (PI), iris and anterior lens capsule may be drawn to anterior vitrectomy cutter which result in large PI and injury to anterior lens capsule [3]. Both Steinwender G [4] and Chung JK [5] agreed that anterior subcapsular cataract was induced during implantation of a ICL with a central hole by cannula irrigation with enforced stream onto the capsule through that hole. This problem can be solved by modified surgical methods, such as the use of IA surgery handpiece.

### Meibomian gland dysfunction and dry eye

ICL surgery may lead to changes in the microenvironment of the eye surface, resulting in meibomian gland dysfunction, or even dry eyes. It was reported by Moshirfar M et al. [6] that dry eye symptoms occurred in 13% of patients on the first day after surgery and peaked at about 29% at 3 months. According to the study of Paul J Dougherty et al., 5.8% of patients undergoing V4 ICL implantation had dry eye after surgery, mainly mild to moderate dry eye, without severe symptom [7]. It was reported that OSDI scores increased, while NIBUT decreased significantly at 1 month after ICL implantation, which restored slowly and did not return to the original level at 3 months after surgeries. Moreover, lid marginal abnormality and Meibomian gland secretion were significantly higher at 1 month postoperatively and returned near the baselines gradually at 3 months postoperatively. In addition, patients who had dry eye symptoms before surgery had significantly worse symptoms after surgery [8, 9]. In another report, the stability of tear film decreased and caused dry eye symptoms of varying degrees in patients in the early stage after ICL surgery, but there was no significant impact on the amount of basic tear secretion. Three months after surgery, the subjective dry eye discomfort symptoms and eye surface tear film stability of patients basically recovered to the preoperative level. However, for patients with high postoperative corneal staining score or OSDI questionnaire score, drugs or artificial tears should be given in time to help repair of corneal epithelium [10].

### Astigmatism

There were few reports about surgical astigmatism of ICL surgery. Kamiya, K. et al. reported that astigmatism caused by ICL surgery with horizontal 3.0 mm transparent corneal incision is 0.45D measured by keratometer and 0.49D by corneal topography. Moreover, its regular astigmatism and will not recover over time [11, 12]. However, it was reported that the magnitude of corneal astigmatism significantly increased in the temporal incision group, but it significantly decreased postoperatively in the superior incision group [13].

### Urrets-zavalia syndrome

ICL-related UZS may be caused by the increase in intraocular pressure postoperative due to viscoelastic agent retention. This condition leads to anterior chamber angle obstruction, potentially promoting ischemia of the root of the iris, resulting in permanent and relative paralysis of the ciliary muscle.

To our knowledge, Kummelil et al. were the first to report an association between UZS and ICL implantation (Poster P85, American Society of Cataract and Refractive Surgery, May 25–29, 2011, San Diego).

In 2015, Al Habash A et al. [14] described the case of a healthy 28-year-old woman who underwent ICL implantation in both eyes for correction of bilateral high myopia with astigmatism, but UZS occurred only in the patient's left eye. On the first postoperative day, the patient developed increased intraocular pressure (IOP) and a fixed, mid-dilated pupil in her left eye. The elevated IOP was corrected within 3 days by medical treatment. However, the pupil remained mid-dilated and nonreactive to both light and accommodative effort during 2 months of follow-up; there was no reaction to pilocarpine (0.125% or 4%) eyedrops.

A similar case was reported by Srirampur et al. [15], who underwent bilateral ICL implantation but developed UZS in only one eye. Because the patient was implanted with a non-toric ICL, rotation of ICL was performed to reduce the high vaulting and optic capture along with single-pass four throw pupilloplasty, and received a successful outcome.

### Iris and ciliary cysts

Gharaibeh et al. reported a 27-year-old patient who developed iris and ciliary cysts after ICL implantation. They suggested that the iris and ciliary cysts originated from corneal or conjunctival epithelial cells that either happened to implant onto the iris during surgery or migrated to the iris via the surgical wound postoperatively [16].

If the cyst is small and asymptomatic, only close follow-up is required, but continued cyst growth may lead to potentially vision-threatening complications such as displacement of ICL.

### Lacquer crack and choroidal neovascularization

Lacquer cracking and choroidal neovascularization are rare complications after ICL implantation, and only a few literatures have been reported [17, 18]. The main pathogenesis is that the posterior sclera of patients with high myopia is thin. ICL implantation leads to intraocular biomechanical abnormalities, which is more likely to cause the rupture of posterior Bruchs membrane and atrophy of the retinal pigment epithelium, resulting in lacquer cracks.

### Retinal hiatus and rhegmatogenous retinal detachment

Retinal hole is one of the complications of ICL implantation. In the process of ICL implantation, acute posterior vitreous detachment can be caused by sudden decompression of the anterior chamber due to the initial establishment of surgical channel or the operator's operation. For young patients with high myopia, the vitreous body and the retina are attached relatively closely, and the acute posterior vitreous detachment can easily pull the retina, resulting in retinal hiatus [19, 20]. Moreover, the crack region generated by ICL implantation would appear in new sites, independent of the patient's existing fissure lesions [21]. If the hiatus appeared within a short period of time after operation and accompanied by internal limiting membrane defect around the hiatus, the prognosis was very poor. If occult progression occurs, surgical treatment results in better best corrected visual acuity [22–24].

Vitreous liquefaction was obvious in patients with high myopia, and the formation of retinal hiatus was more likely to develop into rhegmatogenous retinal detachment. A retrospective study by Martinez-Castillo et al. showed that the incidence of rhegmatogenous retinal detachment after ICL implantation was 2.07%, which occurred on average 29.12 months after surgery, and 68.75% of retinal detachment cases had only one ruptured hole [25]. Rhegmatogenous retinal detachment may occur several hours after ICL or may progress slowly for several months. Most patients can recover good visual quality after treatment, but some patients still cannot recover their visual quality even after therapy [26–31]. However, it was also reported that V4c ICL implantation for high myopia correction does not add RRD risk in the long term [32].

### Endophthalmitis

Endophthalmitis after ICL implantation includes infectious endophthalmitis and aseptic endophthalmitis, with an incidence of about 0.0167% [33].

Infectious endophthalmitis is one of the most serious complications after ICL surgery. Reported pathogenic bacteria include staphylococcus epidermidis, radioactive rhizobia, aspergillus, pseudomonas aeruginosa, cutibacterium acnes, etc. [34–39]. Report of aseptic endophthalmitis after ICL implantation includes Toxic anterior segment syndrome (TASS) and acute aseptic anterior uveitis [40]. TASS is the main cause of aseptic endophthalmitis after ICL implantation, which is induced by the material of pollution of intraocular lavage fluid, talc powder of gloves and so on [41–45].

## Summary and treatment of postoperative complications of ICL

### Night vision symptoms

58% of the patients reported a worsening in night vision symptoms (mostly nonspecific glare and halo or starburst) after surgery, but overall levels of satisfaction were high [46, 47]. The incidence of halo after ICL implantation was 68.8%, and that of starburst was 43.8% [48]. Glare/halo was present in 39% of eyes at 1 month, 32% at 3 months, 24% at 6 months, and improved to 15% of eyes at 12 months. The size of the halo will not change with the luminance levels after ICL implantation, but the incidence will decrease with postoperative time. Halo in about 15.2% of patients and glare in 23.2% postoperatively in the study of Mahfouth A et al. [49], while Dong Hui Lim et al. reported that the incidence of halo and glare was 34% and 26%, respectively. The mismatches of pupil diameter, white to white (WTW) and ICL optical region diameter were positively correlated with halo occurrence, and the high curvature of ICL is one of the significant risk factors for postoperative glare [50]. In addition, postoperative glare occurred in a small number of patients because of laser iridotomy [51].

### Abnormality of arch height

Arch height was positively correlated with anterior chamber depth, pupil diameter, axial length and lens position and negatively correlated with lens thickness [52–54]. At 1 month after surgery, ICL size had the greatest impact on the arch height, followed by horizontal sulcus-to-sulcus diameter (STS), crystalline lens thickness (LT), and vertical STS. The influence of other factors such as white-to-white diameter (WTW) and preoperative spherical equivalent refractive error had no effect [55–58]. Although WTW can indirectly predict STS, the WTW diameter was further away from 11.08 to 12.51 mm, or the ACD was further away from 2.81 to 3.74 mm, the correlation between the STS and WTW diameters was weaker [59].

The causes of postoperative high arch height include the too large ICL relative to the ciliary sulcus, the younger age of the patients, and the higher myopia [60, 61]. Too large intraocular lens relative to the ciliary sulcus is the main influencing factor of high arch height. High crystalline lens rise, and low ICL spherical equivalent were the major risk factors in eyes presenting low arch height [62]. Moreover, arch height also decreased with time and decreased the most from one month to three months after operation [63–76]. But it is also been reported in the literature that the average postoperative arch height will decrease significantly at 6 months after surgery and will remain basically stable thereafter [6]. Schmidinger reported that the arch height was reduced by an average of 28 μm per year [77], while Choi, JH's study showed that the value was 21 μm [78]. Different surgeons have different perceptions of the range of safe arch height, but existing reports suggested that the minimum safe arch height is from 52 to 260 μm [79–82].

In view of the decrease in arch height over time [83], in order to maintain sufficient arch height for a long time (> 10 years), the immediate arch height after operation should be greater than 550 µm [84], and for arch height below 150 µm, immediate removal and/or replacement with a more appropriate ICL should be considered [85]. Additionally, vertical rotation of an ICL may be a less invasive method to treat high arch height in certain cases [86].

With the advent of the microscope-integrated optical coherence tomography (OCT), intraoperative determination of ICL vault using OCT is an effective method for predicting postoperative ICL vault and minimizing postoperative abnormality of arch height that could require surgical retouching [87].

### Abnormal position of ICL

Postoperative position abnormalities include ICL dislocation, ICL rotation and ICL inversion.

ICL dislocation includes subluxation and total dislocation, and the causes involve Improper arch height, residual of viscoelastic agent, and relatively inappropriate size of ICL [88, 89]. There are also some rare predisposing factors, such as the use of specific drugs and trauma. Rui Wang et al. reported a case of postoperative ICL dislocation associated with olanzapine and buspirone. These two drugs both have anticholinergic effects and can cause pupil dilation and ciliary paralysis, which is speculated to make ICL dislocate into the anterior chamber in conjunction with other factors such as face-down ducking and dim-light induced pupil dilation. This case suggests that systemic anticholinergic agents may be a relative contraindication to ICL implantation [90]. Up to now, fourteen cases of trauma-related ICL dislocation had been reported, of which thirteen cases occurred after ocular contusion. Another case was caused by sudden occipital trauma. All of them received ICL repositioning surgery, but postoperative visual acuity recovery varied. Therefore, it is important to educate patients to use protective eyewear during activities with a risk of ocular contusion [91, 92].

Spontaneous rotation is a common postoperative complication. For patients with ICL implantation, spontaneous rotation only affected the arch height of patients to a certain extent, and for patients with TICL, it resulted in a significant loss of visual acuity [93, 94]. The incidence of spontaneous rotation of TICL was about 0.12% [86]. Majid Moshirfar et al. 's study showed that 87% of patients rotated < 5° after TICL implantation and did not require additional intervention [6]. However, it was also reported that approximately 9.375% of patients had a cylinder axis rotation of 10° or greater occurred and required repositioning [95].

Most of the turn-over of ICL occurred during surgery, with an incidence of about 1.8%, and were mostly related to factors such as lens preinstallation, surgical incision, injection process, and anterior chamber depth. Spontaneous turnover after ICL implantation is extremely rare, and only two cases have been reported, and the longest one occurred five years after surgery [96, 97].

### Loss of corneal endothelial cells and corneal decompensation

Direct contact between ICL and corneal endothelial cells and postoperative corneal remodeling are the main causes of corneal endothelial cell loss [98]. Further loss may result in corneal decompensation [99].

It is reported that the lowest rate of endothelial cell loss after non-central hole ICL was 1.1% and the highest was 13.4% within 5 years [100, 101]. Beyond five years after operation, the lowest rate was 4.7% and the highest was 19.75% [102–106]. The lowest rate of endothelial cell loss within five years after ICL with a central hole (V4c ICL) is 0.41%, and the highest is 22.0%, and arch height was the most significant factor for changes in corneal endothelium cell density in V4c ICL [57, 66, 69–71, 73, 75, 76, 107–116].

V4c ICL implantation may have advantages over conventional ICL implantation only in terms of the density in the superior regions, possibly because preoperative laser iridotomies are unnecessary [108]. In addition, the use of viscoelastic agent for V4cICL implantation can effectively protect corneal endothelial cells [117]. However, newer studies had shown that the OVD-free method is safe and efficient for ICL implantation. It can be a safer method of ICL implantation without additional damage to the corneal endothelial cells compared to the standard method and it completely eliminates ophthalmic viscoelastic devices-related complications without causing additional complications [118–124].

### Decreased anterior chamber angle (ACA)/trabecular iris angle (TIA) and anterior chamber depth (ACD)

It was reported that peripheral ACD and ACA after ICL/TICL(V4c) implantation were significantly lower than pre-operative values. The ACD and ACA was significantly narrowed immediately after V4c ICL implantation and the decrease tended to be stable after one month [21, 76, 83]. Variations of peripheral ACD and ACA were greater in eyes after TICL (V4c) implantation compared with identically sized ICL (V4c) implantation and with larger size than smaller size lens implantation [125].

Within one month after ICL implantation, both trabecular iris angle (TIA) and anterior chamber depth (ACD) decreased compared with that before surgery. TIA was reduced by about 7.4–45% postoperatively [105, 126–133], ACD decreased by about 2.4–35% compared with the preoperative level [42, 127, 128, 134, 135].

### High intraocular pressure and secondary glaucoma

High intraocular pressure and secondary glaucoma account for 4.4% of postoperative complications after ICL implantation [136], and the causes include steroid response, viscoelastic agent residue, aqueous misdirection, pupillary block, Iris pigmentation, narrow ACA, etc. [137–139]. The increase in IOP mostly reached its peak at the end of the first postoperative month, and the vast majority of patients can resume normal IOP [137]. Currently, the reported secondary glaucoma after ICL implantation can occur at most 8 years after surgery [68].

Steroid response is one of the most common causes of postoperative high intraocular pressure, accounting for about 64% [136, 138], mainly occurring 1 to 4 weeks after surgery and generally requiring no treatment [140].

High intraocular pressure caused by viscoelastic agent residue can be observed for 24 h. If the patient's intraocular pressure continues to rise and exceeds 35 mmHg, or the symptoms such as nausea and vomiting, swelling pain of the affected eye and headache remain unrelieved, another operation can be considered to remove viscoelastic agent [141].

The incidence of secondary glaucoma caused by postoperative dispersion of pigment, pupillary block and other reasons is between 0 and 5%, and the onset time is variable [100, 142, 143].

The most common complication of late postoperative secondary glaucoma is Pigmentary Glaucoma Syndrome [144, 145]. The ICL implantation can cause pigment deposition in trabecular meshwork and high IOP in the short term [126, 136, 140].

Pupillary block occurs mostly after implantation of ICL without central hole. At present, the central hole ICL can reduce the incidence of pupillary block, eliminates the need for laser peripheral iridectomy preoperatively and obtains satisfactory IOP after surgery [146–151]. In particular, Gonzalez-Lopez [152], Mansoori T [153], Isha G [154] and Frost A et al. [155] had all reported pupillary block glaucoma secondary to central hole obstruction after ICL implantation, which may be related to viscoelastic residue, iris pigmentation, increase in inflammatory factors in aqueous humor and so on. The longest occurrence was 5 years after ICL implantation.

The cause of malignant glaucoma after ICL implantation may be that ICL is too small, which leads to hyperemia of the ciliary body and damage of the suspensory ligament, resulting in relatively poor forward flow of aqueous humor, forcing aqueous humor to flow backward into the vitreous cavity, forming a vicious circle. Moreover, it may also be that viscoelastic agent entered the posterior chamber, resulting in obstruction. Kodjikian et al. [156]. reported that a 23-year-old woman received an ICM 130 V2 myopic phakic intraocular lens (IOL) (Staar Surgical AG) implantation. Three days later, the patient developed malignant glaucoma without pupillary block or choroidal hemorrhage/effusion. As maximum medical treatment failed, rapid secondary surgery was performed with sclerotomy, aspiration in the midvitreous cavity, and removal of the IOL. Almalki S et al. had also reported malignant glaucoma due to aqueous misdirection [137]. Senthil S et al. [138] also reported the occurrence of malignant glaucoma in one eye after ICL implantation, which received the treatment of pars plana vitrectomy and hyaloidotomy. Chanbour WA et al. [157] reported that a V4cICL was placed in the eye of a 31-year-old male patient with high myopia followed by the development of malignant glaucoma. After failing medical treatment for 5 days, a noncomplicated pars plana vitrectomy and anterior hyaloidectomy succeeded in breaking the aqueous misdirection. Furthermore, they suggest that intraoperative miotics may increase the risk of malignant glaucoma after surgery.

Decreased ACA after surgery may cause high intraocular pressure and secondary glaucoma. Ex-PRESS glaucoma filtration surgery might be a safe and effective alternative treatment for intractable glaucoma caused by narrow ACA after ICL implantation [158].

### Occlusion of laser iridotomy

YAG laser iridotomy is required to balance IOP 1 to 2 weeks before implantation of ICL without central hole, which is prone to cause recurrent occlusion of laser iridotomy after surgery, possibly due to surgical trauma or reactivation of static anterior uveitis [159].

### Traumatic prolapse of iris

Jordan W et al. reported the first case of traumatic prolapse of iris after ICL implantation, which may be caused by ocular contusion and high intraocular pressure caused by strong external forces. The sudden increase in intraocular pressure will split the transparent corneal incision that has healed after ICL implantation, and then, the iris will be expelled from the eye [160]. Packer KT et al. had a similar report [114].

### Cataract

Cataract is one of the common complications after ICL implantation [161], and early cataract after ICL implantation is mostly related to surgical trauma, while late cataract is mostly related to contact between ICL and the lens itself [162]. The incidence increases with the passage of postoperative time [68, 74, 77, 84, 142, 163–169]. It has been reported that the incidence of cataract within 10 years after ICL implantation is 12.1% [78] and the risk of cataract formation will increase seven years after ICL implantation [114]. Ohoud Owaidhah et al. also reported a case of bilateral cataract 4 months after ICL implantation for both eyes [170], while the longest reported cataract caused by ICL implantation is 10 years after surgery.

Low arch height is considered to be one of the most important factors in increasing the risk of ICL-associated cataract development [171]. Other factors that increase the risk of ICL-associated cataract include contact between ICL and the lens itself, abnormal aqueous humor circulation on the lens surface after surgery, age greater than 40–45 years old, preoperative diopter greater than -12D to -14D and preexisting lens opacity [53, 80, 82, 84, 172–174]. Miguel J Maldonado et al. reported that the use of miotics after ICL implantation can also cause cataracts, possibly because the lens moves forward while the ICL moves backward during eye adjustment, resulting in rapid contact between them [175, 176]. This does not occur in all patients, but caution is advised against cholinergic agonists in patients with ICL implantation.

The most common type of ICL-associated cataract is anterior subcapsular cataract (ACS). The incidence ranged from 0.3% to 28%, with an average onset time of 3.4 ± 1.9 years after ICL implantation. ACS has a significant positive correlation with age and a significant negative correlation with anterior chamber depth (ACD) [95, 100, 116, 136, 145, 165, 177–179].

Femtosecond laser-assisted cataract surgery (FLACS) combined with ICL extraction has been shown to be a feasible method for patients developing cataracts after the ICL implantation [180–183]. We can also proceed for ICL explanation with cataract surgery and IOL insertion of the affected eye [184].

### Ciliary body detachment and secondary choroidal detachment

Franciscol et al. reported a case of ciliary body detachment and secondary 360-degree choroidal detachment after ICL implantation. After exclusion of other possibilities, it was considered to be caused by preoperative YAG laser iridotomy. The patient received surgical treatment and recovered well [185].

### Suprachoroidal effusion

According to Victoria de Rojas et al., a young man developed suprachoroidal effusion after ICL implantation, possibly related to the postoperative use of acetazolamide, an IOP lowering medication [186].

### Macular edema and macular hole

Julide Canan et al. reported a 33-year-old male patient who developed cystoid macular edema after ICL implantation [187]. Possible mechanisms include continuous friction between ICL and posterior iris surface or ciliary sulcus, posterior vitreous detachment caused by ICL implantation, retinal traction, and surgical reduction after primary surgery. CME after ICL is usually self-limited. For patients with severe conditions, intravitreal injection of NSAID drugs can subside CME, but it is not recommended to use it to prevent CME after ICL implantation.

In the report of Kumar et al. [188], a 28-year-old male was found to have a full-thickness macular hole in his right eye 4 months after bilateral ICL implantation without obvious retinal or vitreous changes, preoperatively. The patient received a treatment of pars plana vitrectomy with inverted internal limiting membrane flap and gained well visual acuity (20/40) with the closure of the hole.

### Microvascular anomalies in myopic retinoschisis

Zhang X et al. [189] reported a case that retinoschisis with supranasal microvascular anomalies of the left eye was found during routine reexamination one year after ICL implantation. The patient had no complaints. The naked eye visual acuity of the left eye was 1.0. This case suggests that the fundus of patients with high myopia without complaint should also be examined in detail and comprehensively. In addition to paying attention to peripheral retinopathy, the posterior pole and middle peripheral retina should be carefully examined, especially the areas that cannot be covered by conventional OCT.

### Thickening of the retinal nerve fiber layer

According to the research of Zhu QJ et al., central retinal thickness (CRT) was significantly higher at one and three months postoperative (all *P* < 0.01). Ganglion cell-inner plexiform layer thickness (GCT) was significantly higher at 1wk, 1, and 3 month postoperative (all *P* < 0.01) [190]. The thickness of the retinal nerve fiber layer (RNFL) increased significantly after surgery, especially in the upper and lower quadrants of the retina, and the thickness of RNFL was not related to the change of the equivalent spherical equivalent refraction before and after surgery or the diopter of the intraocular lens[191].

### Transient reduction in the retinal microvascular network

A statistically significant reduction in microvascular density in the retina and the superficial plexus was found 1 week and 1 month after ICL implantation. However, the microvascular density recovered toward the baseline level 3 months postoperatively [192]. Therefore, patients with pre-existing retinal vascular-related problems should carefully consider whether to perform this operation.

## Conclusion

**Fig. 1:**
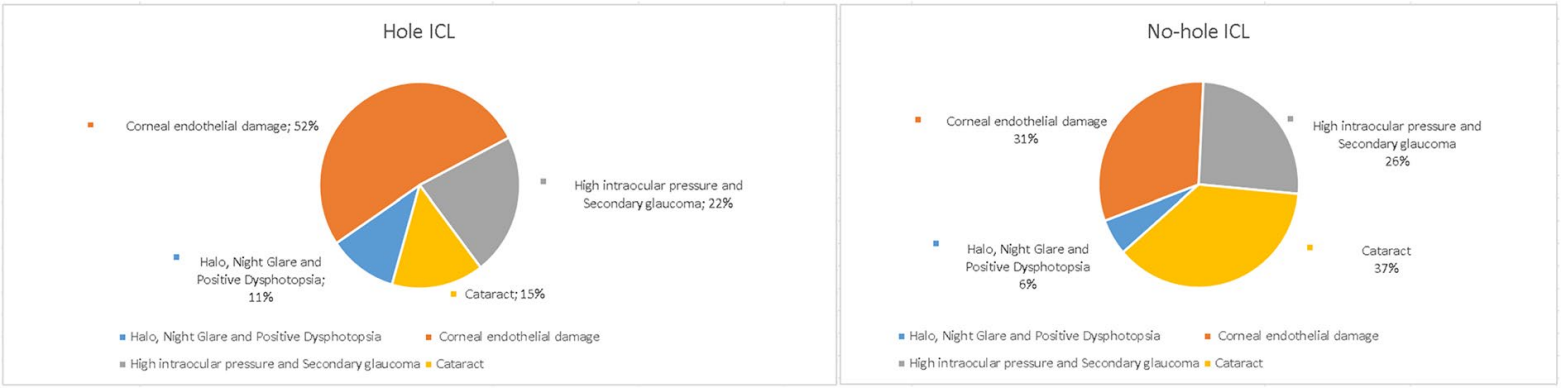
Differences in major complications between hole-ICL and no-hole ICL

There are many kinds of complications related to ICL, but the common intraoperative and postoperative complications mainly include abnormality of arch height, abnormal position of ICL, loss of corneal endothelial cells and corneal decompensation, high intraocular pressure and secondary glaucoma, cataract and night vision symptoms. Compared with ICL without central pore, the incidence of complications such as loss of corneal endothelial cells and corneal decompensation, high intraocular pressure and secondary glaucoma and cataract was relatively lower in central hole ICL, while postoperative complications such as night vision symptoms were obvious. The corresponding proportions are shown in Fig. 1.

## Out look

At present, Evo + ICL implantation is being initially carried out in China. Compared with V4c ICL, Evo + ICL expands the optical area and further provides better guarantee for the improvement of night vision and vision quality of patients [193]. However, Evo + ICL does not solve the problems of spontaneous rotation of ICL, inappropriate arch height and so on. It is hoped that better type of ICL will be designed in the future to solve these problems.
